# Our New York Letter

**Published:** 1892-12

**Authors:** Thomas D. Tuttle

**Affiliations:** 35 West 45th Street; New York


### Correspondence.

### OUR NEW YORK LETTER.

NEW YORK, November 17, 1892.

Though the treatment of typhoid fever by the cold bath is by no means a new subject, some remarks on the results of its use in the New York Hospitals, as well as a brief outline of a method by which it may be given in any private house, and a means of treating hemorrhage from the bowels, may be of interest to some of our readers.

Notwithstanding the fact that cold was used by the ancients in the treatment of fevers, it was not until 1861 that Brand, of Stetten, started it on its new era in the treatment of typhoid fever by introducing the method of which I shall speak. Though the objection is urged against this method that it requires too many attendants and too much paraphernalia for use in private practice, the members of a household are more than glad to thus exert themselves when, after a bath, they see the sufferer with bright eyes and intelligent face, instead of the glassy eyes and delirium disturbed countenance ; and by a method which I shall outline the paraphernalia is reduced to such an extent that it can be had at a moment's notice.

In all cases where the cold bath is used the results are most pleasing, but where the method recommended by Brand is employed, strictly according to his directions, the results are simply astonishing. Up to the time when he introduced this method the mortality in typhoid fever ranged from 20 to 40 per cent., but during seven years of this treatment by Brand, Vogl, and their cogeners, the mortality averaged 2.7 per cent., and in 1,223 consecutive cases treated by them only 12 died, but in these cases his ideas were carried out minutely, and they did not hesitate to give a bath to any case that resembled typhoid because they were not sure it was a correct diagnosis. Vogl, during his forty years experience in the Garrison Hospital at Munich, saw all the different methods tried that have been advanced in this time, but none of them ever reduced the death rate to any marked degree until the cold bath was introduced, when it suddenly fell so low that to-day the statistics are received by many with a question mark.

Any treatment that will so reduce the mortality of a disease, let it be heroic or what not, should have the most careful consideration of the profession and given a thorough trial before it is dismissed, as too many are disposed to do, as impracticable. The words of H. C. Wood should certainly induce the profession to investigate their foundation : "I have no doubt that many persons have died in the United States from typhoid fever whose lives might have been saved by Brand's method if the American medical profession had risen above the opposition of the laity and above their own prejudices."

If the physician is thoroughly convinced that this is the best and safest method of treatment, the laity will follow his directions, but if he says, "I think a cold bath might do the patient some good," the patient will not get one, for it requires the persuasion of self-conviction to induce the friends of the sufferer to give this treatment. The first few baths must be administered by the physician himself, for the complaints of the patient will overrule the judgment of his friends, for until they see the good effects of the bath, it appears to them to be a cruel treatment.

One point insisted on by Brand is that the baths must be begun as early in the disease as possible ; as soon as typhoid fever is suspected start the bathing, and if it does not turn out to be typhoid no harm is done, and in some cases good has resulted ; even in cases where typhoid was suspected, which were really pneumonia, baths have been administered with no bad result. Several cases of this nature have been reported by European writers, and one was presented in the clinics at New York Hospital last winter in which the patient recovered promptly, though he had several baths.

The temperature of the bath should be from 65 to 68 deg. F., should be given every three hours if the patient's temperature is at or above 102.2 deg. F., and continued ten to fifteen minutes. While the patient is in the bath, water at about 55 deg. F. should be frequently poured over his head, and the extremities and body should be rubbed vigorously by attendants, while the patient is encouraged to rub himself, thus causing an alternate contraction and dilatation of the arteries keeping the cold water in constant contact with all parts of the body, and stimulating the superficial nerves.

When the temperature ranges from 101 degs. to 102.2 degs. F., cold packs should be applied to the abdomen at a temperature of 65 degs. F. and changed every half hour if the patient is awake.

The *rationale* of the cold bath is minutely described in a series of papers in the *N. Y. Med. Journ. '89-'90.*

The effect of the cold bath treatment is not simply reduction of the temperature ; indeed this is the least of its effects ; it refreshes the nervous system, deepens the respiration, moistens and cleans the tongue, improves the appetite, steadies and slows the pulse, increases the excretion of urine, and removes stupor and delirium.

When the cold bath method is being followed, no other treatment should be used, except that of feeding the patients. They should be given veal, mutton or chicken broth, which has been allowed to cool and the fat removed, or milk with a little extract of malt in it. Alcoholic stimulants should be given when the condition of the patient demands it, and in most cases, just before he is put in the bath and after he is taken out.

In considering the result of any form of treatment in typhoid fever, we must know the character of the epidemic, for in some epidemics any treatment seems to give good results, while in others, the cases seem to die in spite of all treatment. The work of Vogl, extending over a period of forty years, observing every form of treatment that has been advanced, and especially the cold bath method, furnishes the results of this treatment in every form of epidemic from the mildest to the most severe.

In hospital practice, the results of the cold bath treatment can be justly estimated, for the patients do not present themselves until they are well along in the disease, usually near the end of the week, and one of the requisite points in the treatment is that it be begun early, which can be accomplished much better in private than in hospital practice. Notwithstanding this fact, the results of this method even in the hospitals are very gratifying. In New York hospital during the the year ending Nov. 1st, 1892, there were 76 typhoid patients treated by the cold bath with a mortality of only 5 per cent.

The Brand method was carried out more perfectly in this hospital than in any other in the city, as in most of them they mixed the treatment; in some, giving a bath when the patient seemed to stand the high temperature poorly, and in others, giving baths together with intestinal antiseptics and other medication, but in none have I been able to find as good results as those given above.

Granted that the tub-bath is by far the best method of administering cold in typhoid fever, it has at least two serious disadvantages for use in private practice. First, it is rare that a tub suitable for this purpose can be found in a private house, for it must be a full-sized bath tub that can be moved to the side of the bed, and second, when the patient is delirious he makes a great fuss about being put in the tub, and the members of the household, being unable to appreciate that he is partially unconscious, think he is being treated inhumanly, and the result is the treatment is discontinued, either with the physician's consent, or by a new doctor taking charge of the case. The first of these objections is easily overcome, and the second greatly disminished by the following method, which in want of a better name, I will call the "bed bath."

This method is very simple and the articles necessary to carry it out can be found in every house or at a country dry-goods store. A rubber sheet, large enough to extend well over the sides and ends, is placed over the bed, under the patient, and four blankets are made into rolls and placed under the sheet at the ends and sides of the bed, thus making a rubber bath tub, in which the patient lies, deep enough to about two-thirds cover him. Attendants are at hand with vessels of water at 65 deg. F., and sponges or bunches of gauze with which they apply the water, by a gentle slapping and rubbing, to all parts of the body, until the tub is filled. Pieces of ice, wrapped in gauze to prevent their coming in contact with the patient's body, are placed in the water to keep it at the dedired temperature. This bath is continued for fifteen minutes, when the water is removed by siphon or sponges, the sheet and patient are wiped dry, and the latter wrapped in a linen sheet, and, if shivering, a light blanket is thrown over him. This method is used in one of our best hospitals with cases, where, for any reason it is thought best not to lift them from the bed to the tub, and, though not quite so marked as in the tub-bath, the fall of temperature is very decided, 1.5 deg. F. to 2 deg. F., and the other effects are practically the same as in the tub-bath. There seems to be something less fearful to the patients' minds, in this form of bathing than in the ordinary tub-bath, for they rarely object to it more than to the ordinary sponging with alcohol.

Hemorrhage from the bowels is not a contra-indication to this form of bathing; in fact, it is in cases of hemorrhage that this is most frequently used in the hospital. In cases of severe hemorrhage, it is rare that a bath is called for, as it is the rule for the temperature to fall and not to rise again to the bathing point, though the patient often recovers.

A unique method of treatment in cases of hemorrhage from the bowels, is that of "tieing off" the limbs, now in use in our hospitals with most beneficial effects. This consists in passing an elastic band with a buckle on it, (a piece of suspender will answer admirably), around each of the limbs close to the body. These are tightened sufficiently to check the venous return and yet not obstruct the arterial flow, thus keeping a large amount of blood out of the trunk, and thereby greatly lowering the pressure in the intestines. At proper intervals, to be determined by the condition of the limbs, one band at a time is loosened sufficiently to permit free circulation for about ten minutes, and then tightened again. This is continued for several days, depending on the severity of the hemorrhage.

35 West 45th Street. THOMAS D. TUTTLE.

